# Chemical Reaction and Ion Bombardment Effects of Plasma Radicals on Optoelectrical Properties of SnO_2_ Thin Films via Atomic Layer Deposition

**DOI:** 10.3390/ma14030690

**Published:** 2021-02-02

**Authors:** Pao-Hsun Huang, Zhi-Xuan Zhang, Chia-Hsun Hsu, Wan-Yu Wu, Chien-Jung Huang, Shui-Yang Lien

**Affiliations:** 1 School of Information Engineering, Jimei University, Jimei District, Xiamen 361021, China; ph.huang@jmu.edu.cn; 2 School of Opto-Electronic and Communication Engineering, Xiamen University of Technology, Xiamen 361024, China; 1922031023@stu.xmut.edu.cn (Z.-X.Z.); chhsu@xmut.edu.cn (C.-H.H.); 3 Department of Materials Science and Engineering, Da-Yeh University, Dacun, Changhua 51591, Taiwan; wywu@mail.dyu.edu.tw; 4 Department of Applied Physics, National University of Kaohsiung, Kaohsiung University Road, Kaohsiung 81148, Taiwan; 5 Fujian Key Laboratory of Optoelectronic Technology and Devices, Xiamen University of Technology, Xiamen 361024, China

**Keywords:** atomic layer deposition, tin oxide, plasma radical, oxygen vacancy

## Abstract

In this study, the effect of radical intensity on the deposition mechanism, optical, and electrical properties of tin oxide (SnO_2_) thin films is investigated. The SnO_2_ thin films are prepared by plasma-enhanced atomic layer deposition with different plasma power from 1000 to 3000 W. The experimental results show that plasma contains different amount of argon radicals (Ar*) and oxygen radicals (O*) with the increased power. The three deposition mechanisms are indicated by the variation of Ar* and O* intensities evidenced by optical emission spectroscopy. The adequate intensities of Ar* and O* are obtained by the power of 1500 W, inducing the highest oxygen vacancies (O_V_) ratio, the narrowest band gap, and the densest film structure. The refractive index and optical loss increase with the plasma power, possibly owing to the increased film density. According to the Hall effect measurement results, the improved plasma power from 1000 to 1500 W enhances the carrier concentration due to the enlargement of O_V_ ratio, while the plasma powers higher than 1500 W further cause the removal of O_V_ and the significant bombardment from Ar*, leading to the increase of resistivity.

## 1. Introduction

In the past few decades, tin oxide (SnO_2_), as a promising semiconductor material, has gained significant attention due to the excellent photoelectric properties, such as high transparency, suitable band gap and band structure, and superior electron mobility. In recent works, there are abundant techniques to prepare the SnO_2_ thin films, including magnetron sputter deposition (MSD) [1,2,3,4], thermal evaporation [5], spray pyrolysis deposition [6,7], sol–gel process [8,9,10,11], and chemical vapor deposition (CVD) [12,13,14,15]. However, these technologies still have many shortcomings such as poor coverage, presence of pinholes, and the uncontrolled defects. The atomic layer deposition (ALD) has become a proper candidate to improve the above-mentioned hindrances due to high volatility of precursors, lower process temperature, and pinhole-free deposition at atomic scale. Therefore, the ALD has been widely harnessed in the innovative optoelectronics devices, such as sensors [16], batteries [17], transistors [18], solar cells [19,20], light-emitting diode [20], and flexible electronic devices [21]. At the same time, plasma-enhanced ALD (PEALD) not only enhances the coverage of layer-by-layer at the atomic scale in ALD, but facilitates the production of uniform, dense, and conformal films. Comparable to what has been accomplished for ALD, the challenge for PEALD regards the effects of plasma radicals on the deposition mechanism and the quality of deposited SnO_2_ thin films. With the development of plasma assistance for the deposition, there are an increasing number of articles about high-quality PEALD SnO_2_ films [22,23,24,25,26]. On the other hand, the SnO_2_ thin films have been widely used in many applications such as gas sensors [16], photovoltaics [19,20], and photodetectors [20]. In particular, the SnO_2_ thin films recently demonstrate great potential for the use as electrical transport layer of perovskite solar cells [19,23]. PEALD is a promising thin-film deposition technique owing to its pinhole-free deposition, ability to control thickness at atomic level, and high conformality on 3D substrates. However, the influences of plasma radicals on the surface reactions and film properties are rarely reported. It is known that the photoelectric properties of SnO_2_ thin films greatly depend on the intrinsic defects which are formed during the deposition process. As one of the major defects, some studies reported that the presence of oxygen vacancy defects may lead a trade-off between the loss in optical properties and the gain in electrical properties of the SnO_2_ [7,27,28,29,30], and thus the control of the oxygen vacancy defects depends on applications. It is necessary to acquire a balance between optical and electrical consideration. Owing to the high reactivity, the oxygen gas (O_2_) is one of the most used sources to generate oxygen radicals, assisting the chemical reaction in PEALD process. Accordingly, it is found that PEALD, playing a crucial role in high quality of SnO_2_ thin films, is worth researching deeply because the coordination of oxygen vacancies is related to the intensity of oxygen radicals [24]. Therefore, the target of this study is to optimize the PEALD O_2_ plasma power and investigate its effect on the SnO_2_ thin film properties. This study is helpful for preparing high-quality SnO_2_ to be used in perovskite solar cells.

Here, the preparation for PEALD SnO_2_ thin films at different plasma power from 1000 to 3000 W is studied. Both argon gas (Ar) and O_2_ are ionized to plasma as reactant to react with the used metal precursor of tetrakis(dimethylamino)tin (TDMA-Sn). The effects of radical intensity on the deposition mechanism, optical, and electrical properties of the SnO_2_ thin films are investigated to discuss the relationship between oxygen vacancies and radicals.

## 2. Materials and Methods

### 2.1. Materials and PEALD Process

The SnO_2_ thin films were deposited by PEALD (R200, Picosun, Suzhou, China), where the remote plasma is equipped by inductively coupled coil with radio frequency of 13.56 MHz. The power of the PEALD is purchased from Applied Energy, USA with the reflected power of zero. The Ar (99.999%) and O_2_ (99.999%) mixture is ionized into plasma. The used substrates are glass of soda-lime glass (Asahi, Tokyo, Japan) with a thickness of 0.2 mm and a size of 3 × 3 cm, and p-type silicon wafer with (100). The used precursor of Sn source was TDMA-Sn (C_8_H_24_N_4_Sn, 99.9999%, Nanjing Ai Mou Yuan Scientific Equipment, Nanjing, China) maintained in the bubbler at 50 °C in PEALD process. The Si and glass substrate were cleaned in an ultrasonic bath with deionized water, acetone, and ethanol for 15 min, and then dried with nitrogen gas (N_2_) and in an oven at 70 °C for 30 min. The substrates were placed on the substrate holder in the deposition chamber of PEALD. In the PEALD process, the substrate temperature was kept at 300 °C and the plasma power was varied from 1000 to 3000 W. The first deposition cycle consisted of following steps: the TDMA-Sn pulse of 1.6 s and then the N_2_ (99.99%) purging of 6 s. Then, before the N_2_ purging, plasma exposure of 5 s. The N_2_ of 120 sccm was used as the carrier gas to bring out the TDMA-Sn vapors, which were then diluted by a 400 sccm N_2_ gas before entering the chamber. Afterwards, the deposition chamber was pumped to 10^−4^ Torr before introducing the O/Ar gas mixture. The plasma was generated by the Ar as carrier gas of 80 sccm and O_2_ of 150 sccm with 11 s into the reaction chamber. The N_2_ purging gas thus should not influence the O/Ar plasma.

### 2.2. Characteristic Measurements

**Figure 1:**
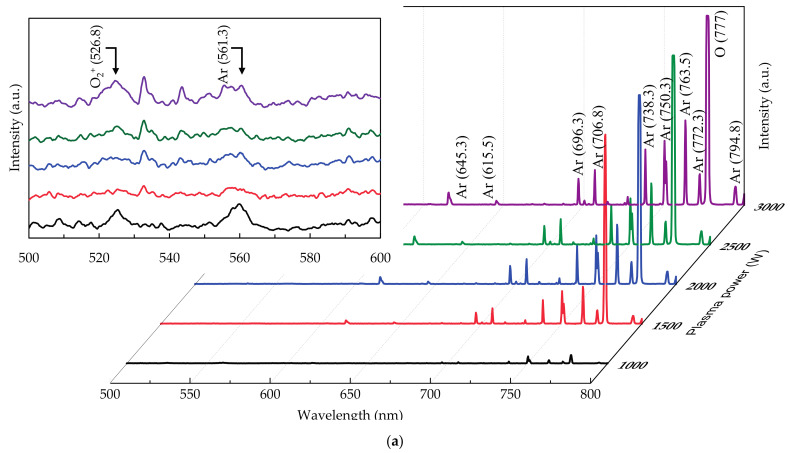

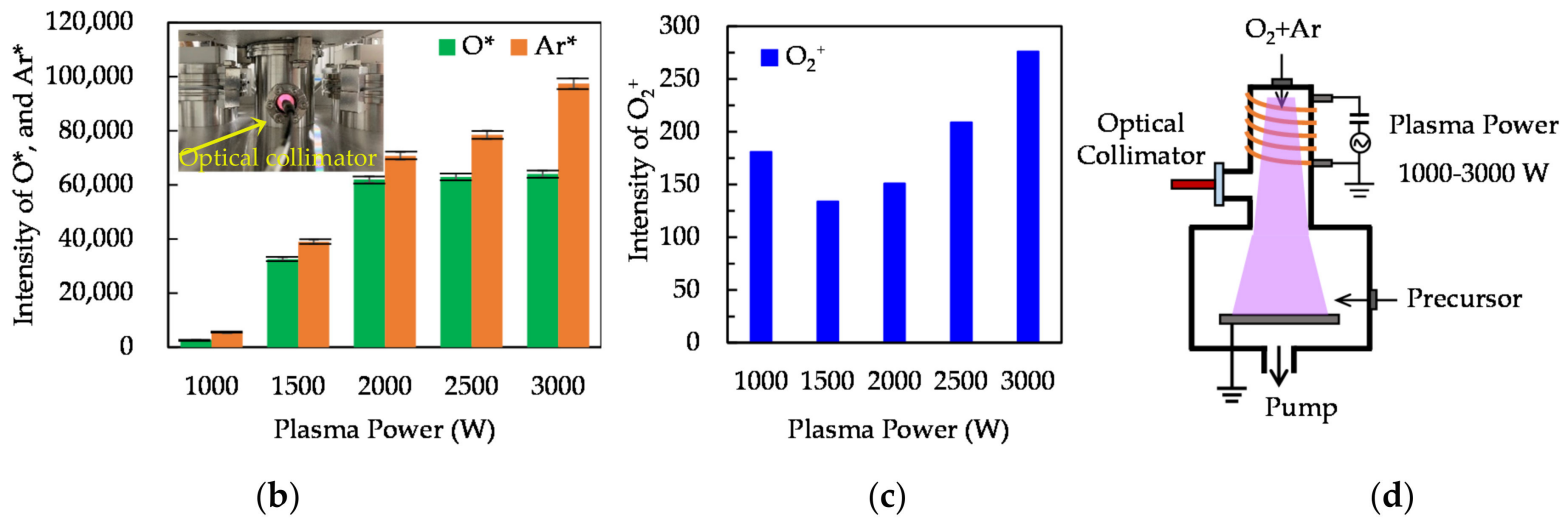
(**a**) Optical emission spectroscopy (OES) spectra, and (**b**) intensity variation of O*, Ar*, and (**c**) O_2_^+^ with increased power from 1000 to 3000 W in plasma-enhanced atomic layer deposition (PEALD) SnO_2_ thin films deposition. The inserted image is the photo of glow discharge at 1500 W. (**d**) The schematic diagram for the ionization of Ar and O_2_ gas in PEALD process equipped with the OES optical collimator.

Five samples were prepared for each plasma power. Both thickness (*d*) and refractive index (*n*) of the SnO_2_ thin films were determined by using an ellipsometer (M-2000, J. A. Woollam Co., Inc., Lincoln, NE, USA) to estimate the deposition rate. The error estimation of the *d* was less than ±2%, indicating a good reproducibility. For the ellopsometric data, the films were prepared on a silicon wafer, and a model consisting of “air, air/SnO_2_, SnO_2_, SnO_2_/silicon” was used, where the SnO_2_ layer was fitted using Drude–Lorentz model. The optical properties of all samples were measured in the wavelength range of 350 to 850 nm via the ultraviolet–visible spectroscopy (MFS-630, Hong-Ming Technology, New Taipei, Taiwan). The plasma radical intensities were obtained by the optical emission spectroscopy (OES, SD2048DL, Verity, Carrollton, TX, USA) from 350 to 800 nm. The peaks at 696.3, 706.8, 738.3, 750.3, 763.5, 772.3, and 794.8 nm are assigned to the Ar* lines [31,32,33,34], while the peak at 777 nm is designated to the O* line [31,32,33,34]. The Ar* lines intensities are summed and illustrated in Figure 1b (together with the O* line intensity). The OES spectra were measured through a quartz optical window, which has very low absorption coefficients in the visible light range and thus has little influence on the measurement. The ratio of oxygen vacancies (O_V_) to oxygen lattice (O_L_) was calculated by the results of peak-differentiating and imitating for the O 1s core level obtained by X-ray photoelectron spectroscopy (XPS, ESCALAB, 250Xi, Thermo Fisher, Waltham, MA, USA). The samples were sputtered before XPS measurement to remove surface contamination. The XPS spectra were calibrated by C 1s (284.5 eV). The peak value of O_V_ was located at 532 ± 0.1 eV and that of O_L_ were located at 531 ± 0.1 eV [24,25,26]. The Sn 3d peak for the films prepared at different plasma powers are added as shown in Figure S1. The Sn 3d curves are deconvoluted into two components of Sn^4+^ and Sn^2+^. The higher binding energy component at ~487.5 eV is attributed to the Sn^4+^, while the lower one at ~486.4 eV is typically assigned to Sn^2+^ [24,25,26]. The electrical properties including the resistivity, carrier concentration, and mobility were conducted by Hall effect measurements (HMS-5000, Side Semiconductor Technology, Xiamen, China) at room temperature. The resistivity of all samples was further illustrated with 4-point probe (T200OA2, Ossila, Sheffield, UK).

## 3. Results and Discussion

### 3.1. Deposition Mechanism

Figure 1a shows the intensity variation of ionized argon (Ar*) and oxygen (O*) at different plasma power by the OES measurement from 500 to 800 nm. The O peak (777 nm) is observed as the plasma power reaches 1500 W, revealing the dramatic increase of intensity. There is another O_2_^+^ peak at 526.8 nm from 500 to 600 nm obtained by 1000 W as shown in the inserted image of Figure 1a. Figure 1b indicates that the Ar* intensity increases continuously with the increased plasma power, contributing to the increase of O* amounts [31,35,36]. The O* intensity increases sharply from 1000 to 2000 W but remains unvarying afterwards. The power of 1000 W is too weak for electrons to obtain sufficient kinetic energy for collision with gas molecules, leading to the lower intensities of Ar* and O*. Therefore, the collision would increase with the rise of power, corresponding to the significantly increased intensities of Ar* and O* at 1500 and 2000 W. This result also shows that the degree of O_2_ ionization reaches maximum at 2000 W. It is reported that the excited energy of the Ar is lower than the dissociation energy of the O_2_. [37]. Furthermore, the Ar also enhances the dissociation of the O_2_. The intensity of O* thus saturates earlier than that of Ar* at increasing plasma power. The photo of glow discharge from Ar* and O* is shown as inserted image of Figure 1b. Figure 1c shows the intensities variation of O_2_^+^ peak obtained at different plasma powers. The lowest OES intensity of O_2_^+^ peak is observed at 1500 W. The overall intensities of O_2_^+^ peak are weaker than Ar* but the O_2_^+^ peak may be considered as a factor of ion bombardment. Thus, the film at 1500 W has the smallest ion bombardment due to the minimal sum of Ar* and O_2_^+^ intensities. The scheme of PEALD reactor with remote plasma and optical collimator is indicated in Figure 1d.

**Figure 2:**
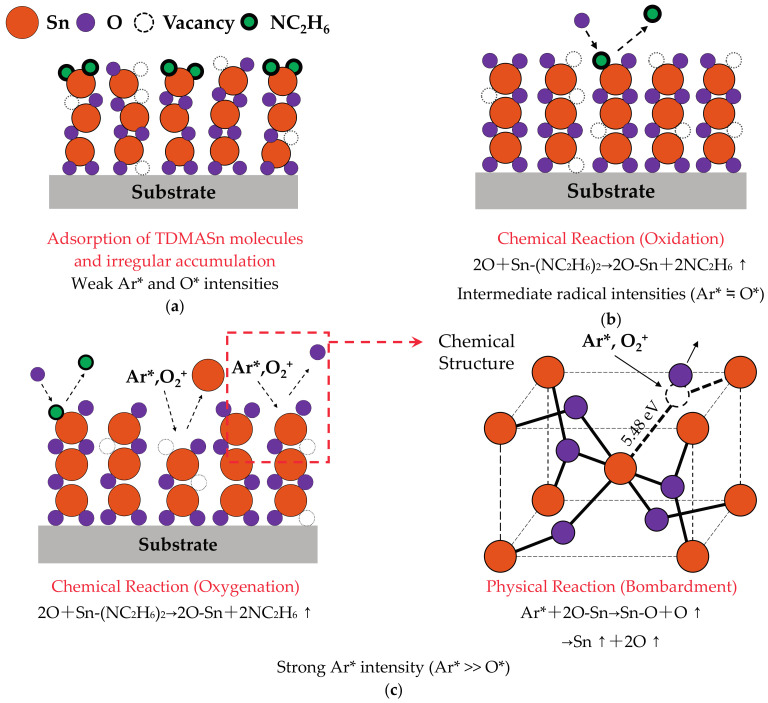
Deposition mechanisms of PEALD SnO_2_ thin films under three kinds of Ar* and O* intensities: (**a**) weak, (**b**) similarly intermediate (Ar* ≒ O*), and (**c**) strong (Ar* >> O*). The inserted image is the chemical structure diagram of films via Ar* and O_2_^+^ bombardment.

Three types of deposition mechanisms of PEALD SnO_2_ thin films are demonstrated in Figure 2: (a) weak Ar* and O* intensities, (b) similarly intermediate radical intensities (Ar* ≒ O*), and (c) strong Ar* intensity (Ar* >> O*). As shown in Figure 2a, corresponding to the deposition at 1000 W, the weak radical intensities do not cause enough bond breaking of precursors and oxidation due to the lower plasma density [31,35,36]. This unsaturated reaction causes the adsorption of precursor molecules and the irregular accumulation of Sn and O atoms. The deposition is in oxygen-deficient condition leading to the growth of oxygen vacancy defects and the loose structure of films [36,38,39]. As shown in Figure 2b, corresponding to 1500 W, the oxidation reaction begins to saturate due to the sufficient intensities of Ar* and O*. At the same time, the deposited structure is denser than other samples owing to the sufficient O* participating the film growth [24,25,36]. The film density usually has a strong correlation with the *n* [23]. The plasma power of 1500 W shows the largest *n*, suggesting the highest film density compared to other plasma powers. The increased Ar* intensity at 2000 and 2500 W may lead to damage on the films surface (although it could be trivial at this stage), inducing the reduced crystal structure. In Figure 2c corresponding to 3000 W, the Ar* and O_2_^+^ peak contributes to the ion bombardment. The much greater Ar* intensity than O* causes serious bombardment and severe structural destruction with the dissociation of Sn-O bonds. This bond breaking occurs when the energy of Ar* is higher than 5.48 eV [40] as shown in the inset of Figure 2c. As a consequence, the induced ion bombardment by too high a power will cause the serious damage to the deposited films although the saturation oxidation reaction is obtained. The deterioration of the film properties due to the strong plasma bombardment can also be found in some researches via MSD [4], and laser-assisted [12] or plasma enhanced CVD [13].

**Figure 3:**
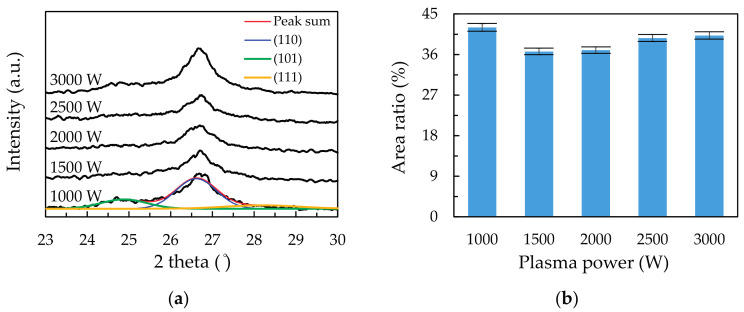
(**a**) Deconvolution of the (110) peak from X-ray diffraction (XRD) patterns for the 2-theta range of 23 to 30°. (**b**) Area ratio of the [(101) + (111)]/total as a function of plasma power.

The XRD measurements have been performed, and the dominant (110) peaks for the films deposited at different plasma power are shown in Figure 3a. It is seen that there are shoulders at the both sides of the (110) peaks, and thus the peaks are further split into three parts. The shoulder peaks at 24.8° and 28.1° correspond to triclinic (101) and (111) Sn_3_O_4_, respectively (JCPDS no. 16-0737) [41,42]. The area ratio of the [(101)+(111)] Sn_3_O_4_ peak to total as a function of plasma power is shown in Figure 3b. The ratio is the lowest at 1500 W and increases when further increasing the plasma power. This in turn means that at high plasma powers, the films have an increased Sn_3_O_4_ formation and the reduced SnO_2_ crystalline structure.

**Figure 4:**
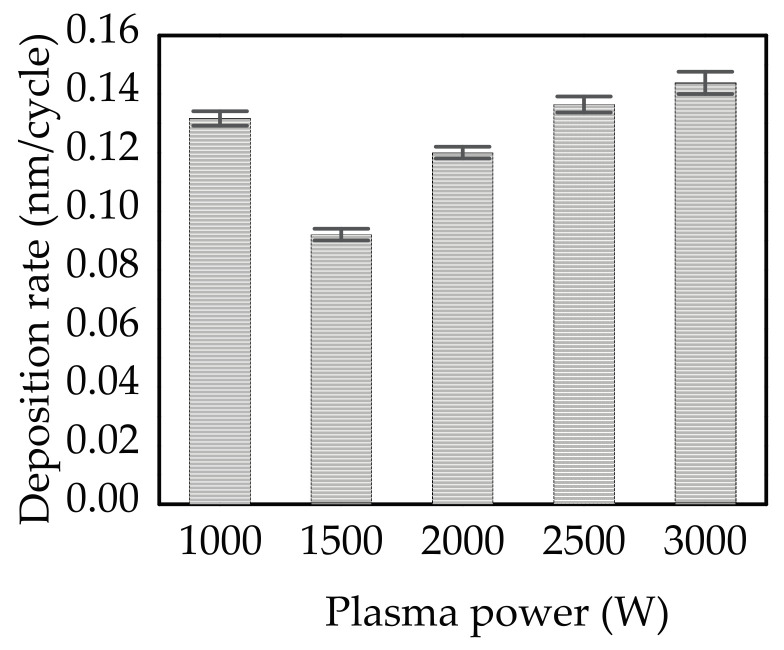
Deposition rate (*R_D_*) of PEALD SnO_2_ thin films deposited at different plasma power from 1000 to 3000 W.

Figure 4 shows the deposition rate (*R_D_*) of PEALD SnO_2_ films as a function of plasma power. The thickness of samples is in the range from 27.6 to 39.6 nm. The *R_D_* is defined by the film growth thickness per cycle. With the increased plasma power, the *R_D_* emerges a V-shaped trend with the lowest value at 1500 W. The high *R_D_* of 1.32 Å/cycle at 1000 W is due to the adsorption of TDMA-Sn molecules and irregular stacking of Sn and O atoms [38,39]. The insufficient oxidation reaction may lead to non-ideal ALD process, where the chemical vapor deposition-like process may occur and lead to a high *R_D_* due to the island growth [43]. In the range of 1500 to 3000 W, the increased *R_D_* from 0.92 to 1.44 Å/cycle is due to the enhanced plasma density [31,35,36] resulting from the increased Ar* intensity and sufficient O*. However, the increase of *R_D_* slows down, owing to the Ar* bombardment. This result can be reflected on the variation of the O_V_/O_L_ ratio as shown later. We have changed the substrate temperature, TDMA-Sn dosing time, and oxygen plasma pulse time for the SnO_2_ deposition. The *R_D_* tends to saturate at 0.145 nm/cycle. In the present study, the plasma powers of 2500 to 3000 W have the *R_D_* of 0.14 to 0.144 nm/cycle, suggesting that the reaction gradually closes to saturation at the plasma power larger than 2500 W as the difference in *R_D_* between 2500 and 3000 W is small (0.004 nm/cycle).

**Figure 5:**
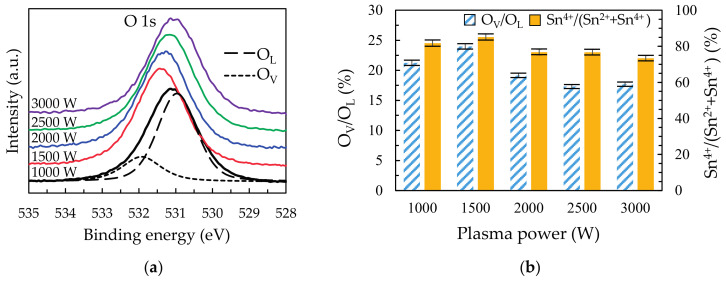
(**a**) The O 1s core level spectra via X-ray photoelectron spectroscopy (XPS) for SnO_2_ thin films deposited at different plasma power from 1000 to 3000 W with the deconvolution of sample at 1000 W and the (**b**) ratio of oxygen vacancies (O_V_) to lattice oxygen (O_L_) and the Sn^4+^/(Sn^2+^ + Sn^4+^).

The high-resolution O 1s spectra are shown in Figure 5a for the SnO_2_ films deposited at different plasma power. The O 1s curves are deconvoluted into two components. The lower binding energy peak at ~531 eV is typically assigned to O^2−^ on the SnO_2_ lattice site (O_L_), while the higher binding energy component at ~532 eV is attributed to the oxygen in the SnO_2_ matrix with O_V_ [24,25,26]. The compositions of the films prepared at different plasma powers are listed in Table 1. In Figure 5b, the O_V_/O_L_ ratio of films shows the opposite trend to R_D_. The enhanced O_V_/O_L_ ratio from 1000 to 1500 W is ~3% (21.2 to 24.1%) due to the sufficient O* which is induced the more saturated reaction. In the range of 1500 to 3000 W, the O_V_/O_L_ ratio decreases due to Ar* bombardment. It is noted that the O* intensity at 2500 and 3000 W is unchanged. As a result, the excess power at 2500 and 3000 W mainly focuses on Ar*, leading to severe bombardment and destruction of films. This result is similar to some studies with high power [4,12,13]. The ratio of Sn^4+^/(Sn^2+^+Sn^4+^) is also revealed in the Figure 5b. The major Sn^4+^ and minor Sn^2+^ states of films are assigned to the bonding of the O_L_ and O_V_, respectively. Thus, the Sn 3d core level spectra via XPS for SnO_2_ thin films deposited at different plasma power from 1000 to 3000 W with the deconvolution of sample at 1000 W are shown in the Figure S1.

### 3.2. Optical Properties

**Figure 6:**
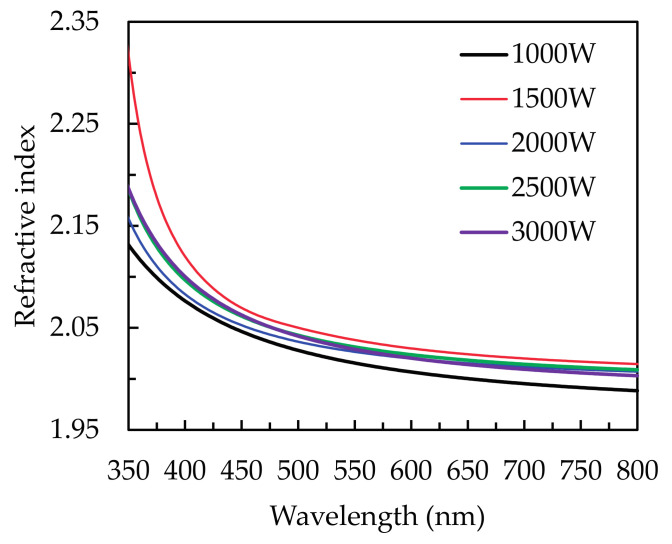
The wavelength-dependent refractive index (*n*) of the PEALD SnO_2_ thin films deposited at different plasma power from 1000 to 3000 W.

The refractive indices (*n*) of all samples are shown as wavelength-dependent functions in Figure 6. The measured substrate is p-type (110) silicon wafers. The highest and lowest *n* values are at the plasma power is 1500 and 1000 W, respectively. The variation of *n* generally has strong correlations to the change of films density [23]. The lowest *n* at l000 W results from the poor film density obtained by the irregular accumulation of Sn and O atoms. The optimized *n* at l500 W is due to the densest structure, attributed to the saturated reaction by sufficient Ar* and O*. Thus, it is comprehensible that the decreased *n* at higher power than 1500 W is caused by bombardment of Ar* [23]. Compared to the literature using MSD [2,3], sol–gel [10], CVD [44], and thermal ALD [19,20,21], PEALD possess a great ability to deposit high-quality SnO_2_ thin films. The refractive indices of SnO_2_ thin films prepared by MSD, thermal evaporation and sol–gel process are 1.96, 2.12, and 2.07, respectively, at the wavelength of around 600 nm [4,5,10]. These values are similar to the values in the present study.

**Figure 7:**
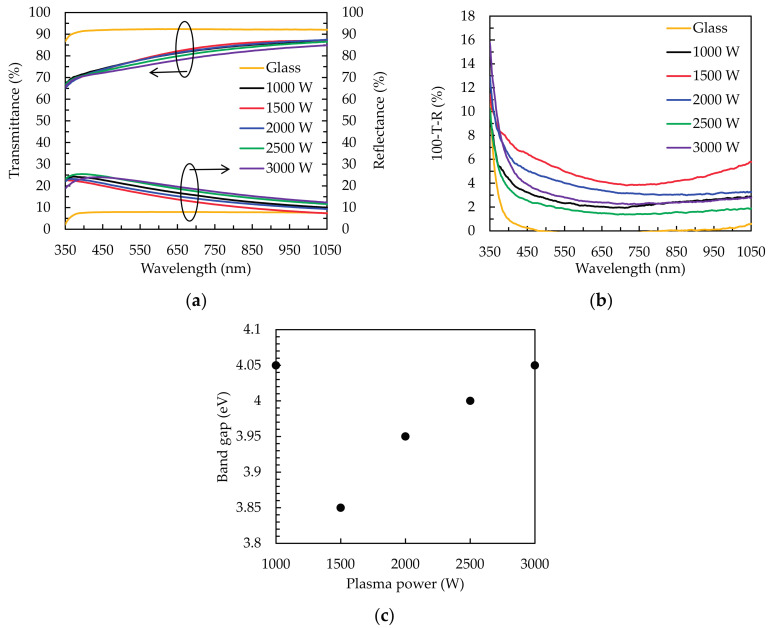
(**a**) Transmittance and reflectance spectra, (**b**) optical loss spectra, and (**c**) band gap (Eg) of the PEALD SnO_2_ thin films obtained by different plasma power.

Figure 7a shows the transmittance and reflectance spectra of the SnO_2_ thin films deposited on the glass substrate at different plasma power. The sample of 1500 W exhibits the highest transmittance of ~80.9% and the lowest reflectance of ~14.3%. Furthermore, the variation of transmittance spectra reveals an inverse correlation to that of reflectance spectra. This change of transmittance spectra is mainly attributed to the reflection conditions caused by different *n*. Particular, at the short wavelength around 400 nm, only the reflectance changes and its trend reveals the red-shift phenomenon after 1500 W. The reason is attributed to the variation of *n*. The optical loss spectrum has estimated via the calculation of 100-*T*-*R* as shown in Figure 7b. The trend of optical loss illustrates the highest and lowest values of ~5% (1500 W) and ~2% (1000 W), respectively. This result is attributed to the consequences of light scattering induced by O_V_ defects [45]. Another possible reason is that the slight absorption of free carriers in O_V_ defects, leading to the narrowing of band gap (Eg) [46,47]. The Eg of the SnO_2_ thin films is determined by Tauc’s plots using the following equation:

αhν = A(hν − Eg)^1/n^, (1)

where α is the absorption coefficient, hν is the energy of incident light, and A is the proportionality constant [23,26]. The value of exponent n is 2 for direct band gap materials, respectively. Figure 7c shows the dependence of the Eg on the plasma power. The evaluated Eg of the samples varies from 3.8 to 4.1 eV with the V-shaped trend, being the contrary variation of O_V_/O_L_ ratio. The minimum value of Eg thus occurs at 1500 W. In addition, the unique distinction for the narrowest Eg at 1500 W is probably owing to the introduction of doping shallow energy level of O_V_ under the conduction band [48]. We have measured OES spectra for the plasma powers of 1430, 1440, and 1450 W to estimate the minimum value of Eg. It is found that the intensities of Ar* and O* at 1430 and 1440 W are weak (similar to that at 1000 W), while the OES spectrum at 1450 W shows the dramatically increased O* intensities as similar to the case of 1500 W. The Eg of sample at 1450 W is measured as 3.86 eV, which is similar to that of 1500 W. For the power higher than 1500 W, the increase of Eg is due to the decrease of O_V_ and optical loss. On the other hand, the *d* of sample is 52.7, 36.8, 48, 54.6, and 57.5 nm with a total of 400 PEALD cycles for the plasma power of 1000, 1500, 2000, 2500, and 3000 W, respectively. The relationship between the *d* and transmittance (T) is given by T = exp(−α*d*). The reflectance is known to be influenced by both of the *d* and *n*. The optical loss is thus also affected by the *d*. The highest optical loss of the thinnest film at 1500 W indicates a high α.

### 3.3. Electrical Properties

**Figure 8:**
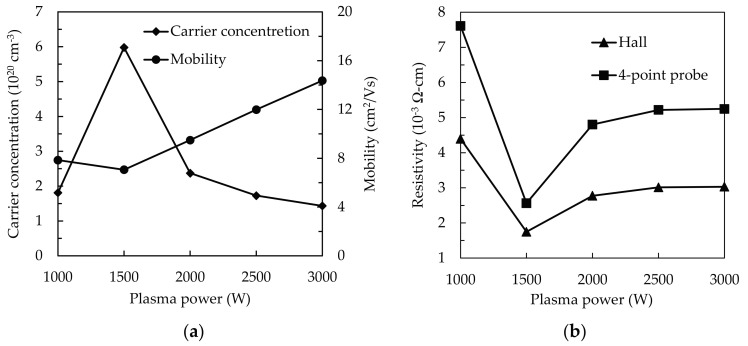
The (**a**) variation of carrier concentration (*Ne*) and mobility (*µ*) and (**b**) the results of resistivity (*ρ*) via respective hall and 4-point probe measurement for the SnO_2_ thin films with the increased plasma power from 1000 to 3000 W.

Figure 8a shows the measurements of carrier concentration (*Ne*) and mobility (*µ*) for SnO_2_ thin films on a glass substrate with the increased plasma power. We observe n-type conductivity for all films. The *Ne* curve sharply increases from 1000 to 1500 W, but dramatically drops from 1500 to 2000 W and then gently decreases. Similar trends have been investigated by some researches [22,24,25]. The increased *Ne* at 1500 W may be ascribed to the increased O_V_/O_L_ ratio as oxygen vacancies have been evidenced as one of the main origins of *Ne*. The subsequent decrease in *Ne* should be related to the suppression of O_V_. As we know, the O_V_ can be acted as the donor doping in metal oxide to the maintaining of charge neutrality. On the contrary, the *µ* shows an upward trend with the increased plasma power. The slight decrease of *µ* at 1500 W is 7.06 cm^2^/Vs with the highest *Ne* of 5.98 × 10^20^ cm^3^. This result can be attributed to the carrier scattering, leading to the inverse relationship between *Ne* and *µ*. As a result, the resistivity (*ρ*) measured by Hall effects and 4-point probe methods indicates an opposite trend with *Ne* as shown in Figure 8b. Lowest *ρ* values of 2.56 × 10^−3^ (4-point probe) and 1.48 × 10^−3^ Ω-cm (Hall-effect) at 1500 W. Then, *ρ* increases from 1500 to 2000 W and shows minor change in the range from 2000 to 3000 W owing to the reduction of O_V_ defects, resulting from the bombardment of Ar*.

## 4. Conclusions

In this paper, the SnO_2_ thin films with high-quality are prepared by PEALD with Ar/O_2_ mixture gas as reactants at different plasma power from 1000 to 3000 W. The radical intensity obtained by OES mainly affects the bond breaking of precursors and the arrangement of Sn and O atoms, suggesting that the Ar* intensity increases with increasing plasma power to induce the increase of O* intensity and even the Ar* bombardment. The immutable change of O* intensity from 1500 W to 3000 W confirms not only the decreased O_V_/O_L_ ratio from the highest 24.1 to 17.1%, but also the decreased films density by Ar* bombardment. The optimized *n* (higher than 2) with better transmittance and reflectance is obtained at 1500 W. The adsorption of precursor molecules and irregular accumulation resulting from insufficient radical intensity of Ar* and O* are the main reason for the higher *R_D_* of 1.32 Å/cycle at 1000 W. Despite the higher value of 4.05 eV is owing to the quantum size effects at 1000 W, the results of band gap also match the variation of optical loss curves due to the light scattering from O_V_ defects. Besides, the power at 1500 W has the lowest *ρ* of 1.48 × 10^−3^ Ω-cm due to the highest *Ne* of 5.98 × 10^20^ cm^3^ and lowest *ρ* of 1.48 × 10^−3^ Ω-cm. The deposition mechanisms and the relationship between the plasma radicals and film properties presented in this study are expected to be helpful for the deposition of high-quality PEALD SnO_2_ films with excellent photoelectrical properties.

## Figures and Tables

**Table 1:** Elemental composition of SnO_2_ thin films prepared at different plasma power.

Plasma Power (W)	Composition		Atomic Ratio [O/Sn]
	O (%)	Sn (%)	
1000	64.2	35.8	1.79
1500	64.2	35.8	1.79
2000	64.1	35.9	1.79
2500	64.0	36.0	1.78
3000	64.2	35.8	1.79

## Data Availability

Data sharing is not applicable to this article.

## Supplementary Materials

The following are available online at https://www.mdpi.com/1996-1944/14/3/690/s1, Figure S1: The Sn 3d core level spectra via X-ray photoelectron spectroscopy (XPS) for SnO_2_ thin films deposited at different plasma power from 1000 to 3000 W with the deconvolution of sample at 1000 W.
