# Supplementary material for: Chemical Reaction and Ion Bombardment Effects of Plasma Radicals on Optoelectrical Properties of SnO2 Thin Films via Atomic Layer Deposition
Source: Materials (Basel). 2021 Feb 2;14(3):690. doi: 10.3390/ma14030690 (PMC7867222; doi:10.3390/ma14030690)
Supplement: Supplementary file 1 [file materials-14-00690-s001.pdf]

materials

MDPI

Article

Chemical Reaction and Ion Bombardment Effects of Plasma Radicals on Optoelectrical Properties of SnO_2 Thin Films via Atomic Layer Deposition

Pao-Hsun Huang [1], Zhi-Xuan Zhang [2], Chia-Hsun Hsu [2], Wan-Yu Wu [3], Chien-Jung Huang [4,*] and Shui-Yang Lien [2,3,5,*]

1 School of Information Engineering, Jimei University, Jimei District, Xiamen 361021, Fujian, China; ph.huang@jmu.edu.cn
2 School of Opto-Electronic and Communication Engineering, Xiamen University of Technology, Xiamen 361024, China; 1922031023@stu.xmut.edu.cn (Z.-X.Z.); chhsu@xmut.edu.cn (C.-H.H.)
3 Department of Materials Science and Engineering, Da-Yeh University, Dacun, Changhua 51591, Taiwan; wywu@mail.dyu.edu.tw
4 Department of Applied Physics, National University of Kaohsiung, Kaohsiung University Road, Kaohsiung 81148, Taiwan; chien@nuk.edu.tw
5 Fujian Key Laboratory of Optoelectronic Technology and Devices, Xiamen University of Technology, Xiamen 361024, China
* Correspondence: chien@nuk.edu.tw; sylien@xmut.edu.cn

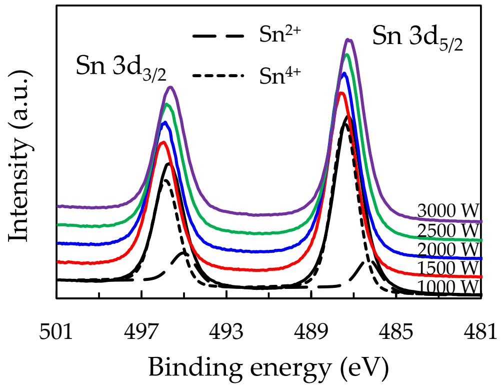

Figure S1. The Sn 3d core level spectra via X-ray photoelectron spectroscopy (XPS) for SnO_2 thin films deposited at different plasma power from 1000 to 3000 W with the deconvolution of sample at 1000 W.

The XPS Sn 3d peaks for the films prepared at different plasma powers are added as shown in Figure S1. The Sn 3d curves are deconvoluted into two components of Sn^{4+} at the binding energy of 487.5 eV and Sn^{2+} at 486.4 eV. The major Sn^{4+} and minor Sn^{2+} states of films are assigned to the bonding of the O_L and O_V, respectively. The area ratio of $Sn^{4+}/(Sn^{2+}+Sn^{4+})$ has been added to the Figure 5b.
